# A New Dimension in Documenting New Species: High-Detail Imaging for Myriapod Taxonomy and First 3D Cybertype of a New Millipede Species (Diplopoda, Julida, Julidae)

**DOI:** 10.1371/journal.pone.0135243

**Published:** 2015-08-26

**Authors:** Nesrine Akkari, Henrik Enghoff, Brian D. Metscher

**Affiliations:** 1 3rd Zoological department, Natural History Museum Vienna, Burgring 7, 1010, Vienna, Austria; 2 Natural History Museum of Denmark, Universitetsparken 15, DK-2100, København Ø–Denmark; 3 Department of Theoretical Biology, University of Vienna, Althanstrasse 14, 1090, Vienna, Austria; Monash University, AUSTRALIA

## Abstract

We review the state-of-the-art approaches currently applied in myriapod taxonomy, and we describe, for the first time, a new species of millipede (*Ommatoiulus avatar* **n. sp.**, family Julidae) using high-resolution X-ray microtomography (microCT) as a substantive adjunct to traditional morphological examination. We present 3D models of the holotype and paratype specimens and discuss the potential of this non-destructive technique in documenting new species of millipedes and other organisms. The microCT data have been uploaded to an open repository (Dryad) to serve as the first actual millipede cybertypes to be published.

## Introduction

Since the time of Linnaeus, approximately 1.5 million new species have been described, which–despite the widespread desire to know the species with which we share our planet–accounts for only a modest fraction of global species diversity. Taxonomists are therefore constantly looking for methods that could accelerate the pace of species discovery and enhance conventional description methods, but only a few attempts have been made to employ modern technologies in myriapodology. Some of the breakthroughs involve next-generation molecular techniques. This emerging discipline, which involves full genome or full transcriptome datasets, owes its existence to the revolutionizing progress in DNA sequencing technology, or next-generation sequencing, supplemented by a simultaneous maturation of bioinformatics. Related advances in phylogenomics, formerly restricted to the biomedical field and genetic model organisms, have now forged the way to help answering questions related to evolutionary biology and overcome the gaps resulting from using single genes in most phylogenetic reconstructions e.g. [1], [2], [3]. Cybertaxonomy is also enjoying healthy progress, including novel methods for species illustration [4], [5], [6] and interactive identification keys, e.g. [4], [7].

Taxonomic descriptions depend, more than any other discipline, upon illustrations. From the earliest taxonomic treatments, species descriptions have nearly always been accompanied by varied kinds of visual representations, which are vital to convey information about the morphology and character states described to distinguish species. The importance of digital imaging in taxonomy has been emphasized in more than one paper, and its role in enhancing species description has been universally recognized; see [8], [9]. Fisher and Dawling listed and described a number of modern and fairly accessible illustration methods used in classical taxonomy [10], while Walter et al. gave an account of the major advances in illustrating techniques from conventional to high throughput technologies that might be capable, when suitably employed, of delivering unprecedented insights on different biological aspects [11].

Line drawings are certainly universal in taxonomy, and were the exclusive medium of illustration in earlier works. Technical limitations did not impede other forms of illustration, like the impressive paintings of Berlese in 1882 [12] or the avant-gardist drawings of Latzel [13], who was the first to recognize the importance of the gonopods for identification of species of Diplopoda in 1884. However, older descriptions usually consisted of short diagnoses accompanied by drawings, many of which are alone unreliable for species identification. This represents a major impediment in taxonomy today, especially in the absence or inaccessibility of type material. Drawing is necessarily a very subjective exercise: even when excellently performed it may still convey erroneous information, and this has led to taxonomic problems on a number of occasions. Several examples exist in the myriapod literature, but among the most striking ones is the case demonstrated by Hauser [14] on the genus *Craspedosoma* Leach, 1814, which revealed that out of the 11 subspecies and 100 varieties described by Verhoeff [15] only 9% are valid while the rest are due to observation errors. Lewis (2009) gave a different example [16], in which a good illustration of Brolemann [17] did convey a useful taxonomic detail overlooked by the author himself and which was depicted later in a subsequent work of Verhoeff [18]. Great myriapod taxonomists are not necessarily great draftsmen, while some of the most impressive drawings published are the artwork of professional illustrators, even in present times.

Scanning electron microscopy had already been introduced in the mid-seventies in myriapod taxonomy when in 1977, Shear presented some peculiar surface structures that he noticed on a few species of Polydesmida [19]. Two years later, Enghoff included SEM images of the mandibles of two newly described species [20], [21]. The same author was perhaps the first to use SEM to represent sexual characters and genitalia [22], a method which has since then become recurrent in myriapod taxonomy and continues today to be used to convey information about minute structures and details, which are most often impossible to represent by line drawings or by verbal descriptions (e.g. [23], [24], [25], [26]).

The use of standard microphotography has been very limited in species descriptions of myriapods, even though it has been employed since the works of Loomis, who presented the habitus of some species of callipodidans and cambaloids (Diplopoda) [27], [28]. It is true that conveying information from a complex three-dimensional structure via photographs can be very difficult and often frustrating, as it can be close to impossible to show in a single shot all relevant details, which occur in different focal planes and are not visible from the same angle. However, the continual progress of photography and computational processing has opened a wide range of possibilities for biological applications; in particular, focal stack imaging can provide excellent pictures of external morphology including sexual characters and can depict additional information including the natural color of the structures, which neither drawing nor SEM allows (also see [29]). Therefore, the use of photomicrographs has become a common complement to species descriptions, and many authors now combine drawings, photomicrographs, and electron micrographs (e.g. [24], [30], [31]). Interactive images have only begun to appear very recently. For example rotational SEM images constructed by combining sharply detailed SEM micrographs into interactive models were used to illustrate millipede gonopods in species descriptions and interactive keys [4], [6].

X-ray microtomography (microCT, or XRM) represents one of the most powerful tools for generating multidimensional and interactive illustrations as well as one of the most useful non-destructive imaging techniques in systematic studies. However, the use of such microCT images to generate 3D models for myriapod systematics has been very limited so far. Stoev et al. included a single dataset of microCT images of the centipede *Eupolybothrus cavernicolous* in their description of the species [5], while Blanke and Wesener used microCT to depict new and potentially useful characters for millipede phylogeny [32]. On the other hand, microCT is gaining popularity for morphological studies of various arthropods (e.g. [33], [34], [35]) and has for example served to investigate functional morphology of millipede genitalia during copulation [36].

Faulwetter et al. emphasized the potential of microCT as an essential tool in today’s taxonomy and further stressed on the importance of the method in revealing a wide range of useful characters in the study of Polychaeta [37]. The same authors also discussed the notion of a cybertype (also called an e-type or virtual type) and the importance of volumetric datasets of an actual type specimen. About same time, the use of microCT in taxonomy was extended to other animal groups such as Oligochaeta [38] and Arachnida [39] whereas it has seen very limited use in myriapod systematics.

In this work we demonstrate the use of microCT in describing new species, and we present for the first time 3D digital models of the type material of a recently discovered millipede species, *Ommatoiulus avatar* **n. sp.** Our results illustrate the potential of this nondestructive imaging technique in depicting male and female genitalia and other anatomical structures, including both chitinous and soft tissue morphology. This species stands as the first millipede to be described using, in addition to conventional taxonomic treatments, volumetric data and 3D models for the male holotype and a female paratype, these data serving as cybertypes.

## Material and Methods

All specimens were preserved in 70% ethanol. Measurements were made using a Nikon DS-Fi1 camera mounted on a Leica MZ125 stereomicroscope. Microphotographs were taken using the same equipment or with a Leica digital camera M205A mounted on a stereomicroscope Leica DFC 420. For scanning electron microscopy, parts of the specimens were transferred to 96% ethanol and then to acetone, air-dried, mounted on aluminum stubs, coated with platinum/palladium, and examined in a JEOL JSM-6335F scanning electron microscope. All images were processed and edited in Adobe Lightroom 5 and Adobe Photoshop CS6 and assembled in Adobe InDesign CS6.

For microCT imaging, the male holotype and a female paratype were dehydrated in 96–100% ethanol and scanned in ethanol in small sealed microcentrifuge tubes using an Xradia MicroXCT-200 system (www.xradia.com) with a micro-focus tungsten source (Hamamatsu L9421-02) and switchable scintillator-objective lenses. Images were made with source settings of 60kV anode voltage and 4W nominal power, with source-object-detector distances adjusted to give the desired field view for each sample. The acquisition and reconstruction parameters for each scan are detailed in the metadata files archived with the cybertype image data. Projection images were taken every 0.2° over a half-rotation (180° plus the beam cone angle; [40]). Each specimen was scanned again after staining with 1% (w/v) iodine in absolute ethanol overnight or longer [41]. After returning the samples to 70% ethanol, the iodine washed out.

Tomographic sections were reconstructed with pixel sizes and slice thicknesses of 1.7, 2.0, or 2.5 μm (isotropic voxels) and 8-bit depth using the Xradia software (XMRecontructor, v. 8.1). The window and level values for the images were chosen in the reconstruction to include the complete brightness range of the samples, and these values were not adjusted further in the cybertype image stacks. The reconstructed tomographic images were exported as 8-bit TIFF image stacks (no compression and retaining the original pixel values), and reoriented, cropped and resliced parasagittally using Fiji [42], then saved as grayscale PNG image stacks (libpng.org/pub/png/). For archiving, the stacks were down-sampled slightly in Fiji (using the Image > Scale function with bicubic interpolation) from voxel sizes of 1.7, 2.0, and 2.5 μm to 1.9, 3.0, and 3.4 μm respectively, in order to bring the file sizes down to approximately 1 GB for each stack. This resulted in no discernable change in image quality.

The taxonomically important structures were segmented in Amira 5.6, and these were highlighted in the volume renderings using selected colormaps and adjusted opacities (not as surface renderings, which eliminate much of the significant 3D information).

The electronic edition of this article conforms to the requirements of the amended International Code of Zoological Nomenclature, and hence the new name contained herein is available under that Code from the electronic edition of this article. This published work and the nomenclatural acts it contains have been registered in ZooBank, the online registration system for the ICZN. The ZooBank LSIDs (Life Science Identifiers) can be resolved and the associated information viewed through any standard web browser by appending the LSID to the prefix "http://zoobank.org/". The LSID for this publication is: urn:lsid:zoobank.org:pub:A517E3F5-A09B-4523-B243-FAC421568898. The electronic edition of this work was published in *PLOS ONE* (eISSN-1932-6203) and has been archived and is available from PubMed Central and the LOCKSS digital repository.

Ethics statement: No ethical issues were involved in this research.

Repository acronyms: NHMW–Naturhistorisches Museum Wien, Austria; ZMUC–Natural History Museum of Denmark, Zoological Museum, University of Copenhagen.

For terminology, we follow Akkari and Enghoff (2012).

## Results

### Taxonomy

Order Julida Brandt, 1833
Family Julidae Leach, 1814
Tribe Schizophyllini Verhoeff, 1909
Genus *Ommatoiulus* Latzel, 1884

*Ommatoiulus avatar* Akkari and Enghoff **n. sp.** *urn*:*lsid*:*zoobank*.*org*:*act*:*6CE666C0-56E2-402C-8EB1-A1907C9309EC*

Figs 1–11; S1–S8 Videos

**Fig 1:**
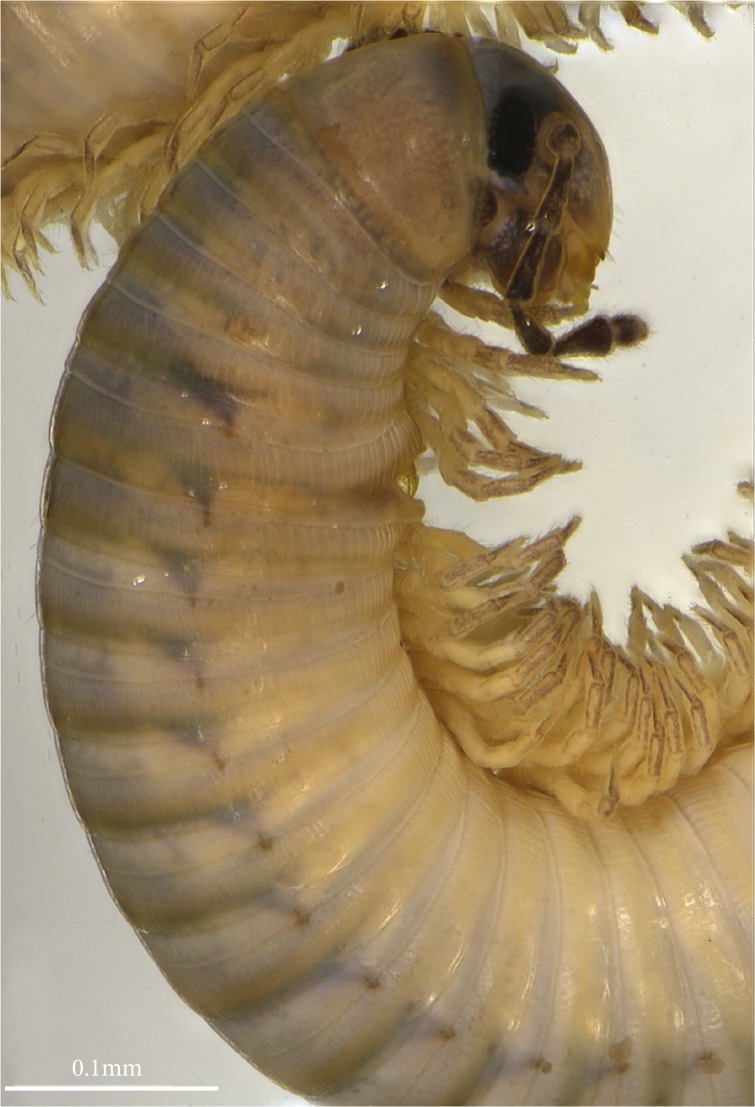
*Ommatoiulus avatar* n. sp., male paratype. Photograph of head and anterior-most rings.

**Fig 2:**
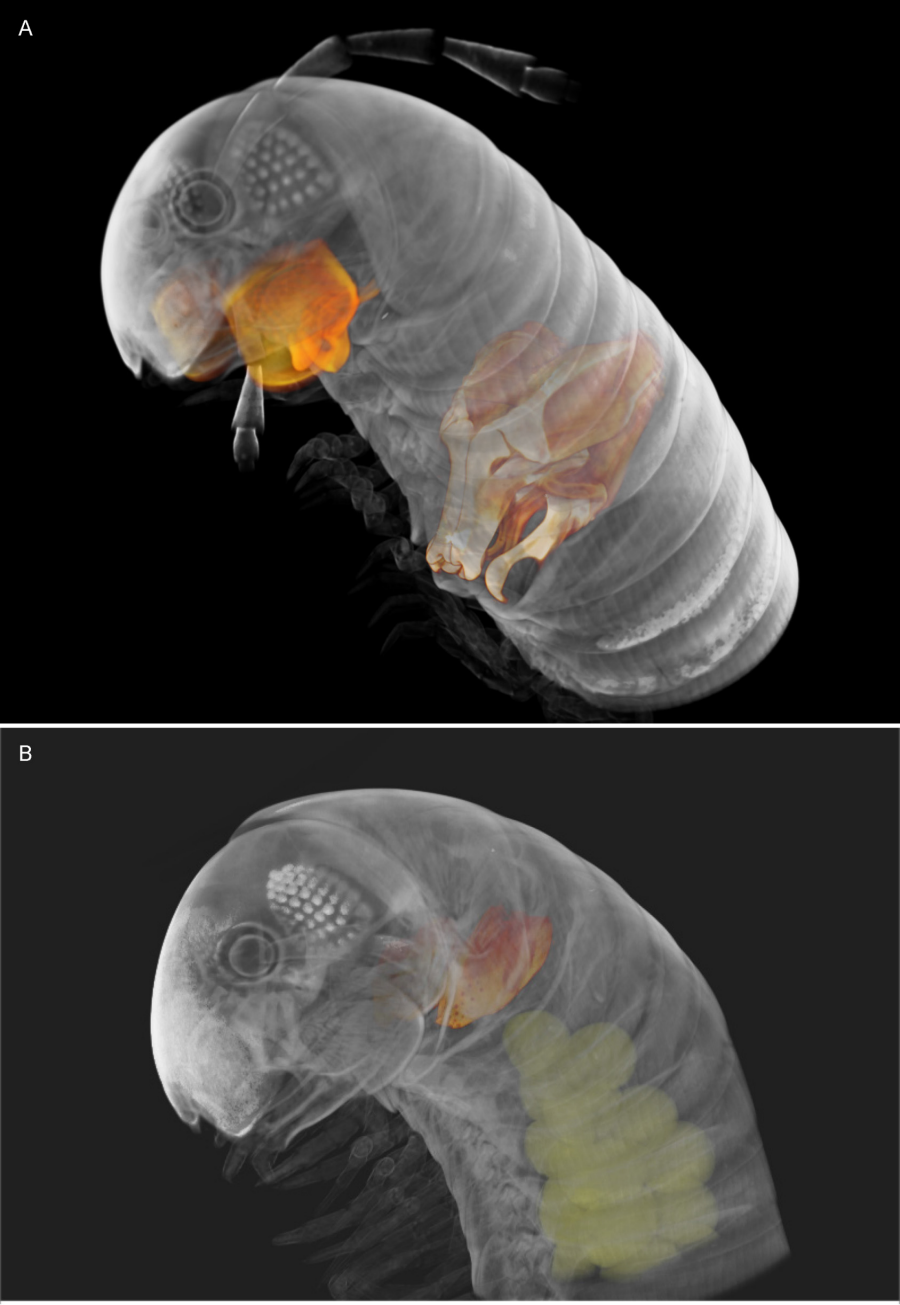
*Ommatoiulus avatar* n. sp. Volume rendering highlighting genitalia and other sexual characters in the cybertypes. (A) Holotype male with highlighted gonopods, first pair of legs and mandibular stipites. (B) Paratype female with highlighted vulvae and eggs. Images not to scale.

**Fig 3:**
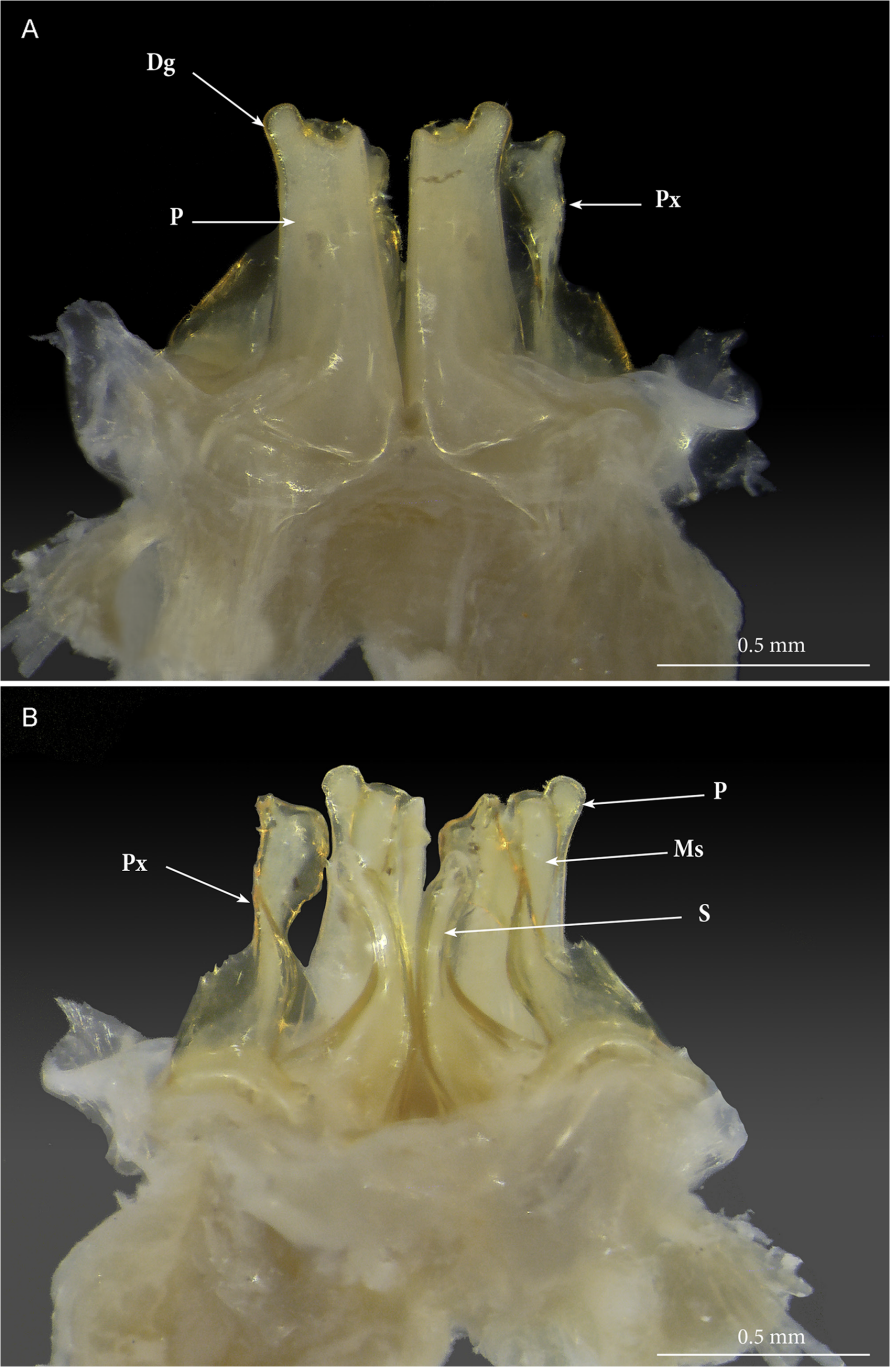
*Ommatoiulus avatar* n. sp., male paratype. Photographs of dissected gonopods in toto. (A) anterior view; (B) posterior view. **Abbreviations**. **Dg**. digit-shaped process; **Ms**. mesomerite; **S**. solenomerite; **P**. promerite; **Px**. paracoxite.

**Fig 4:**
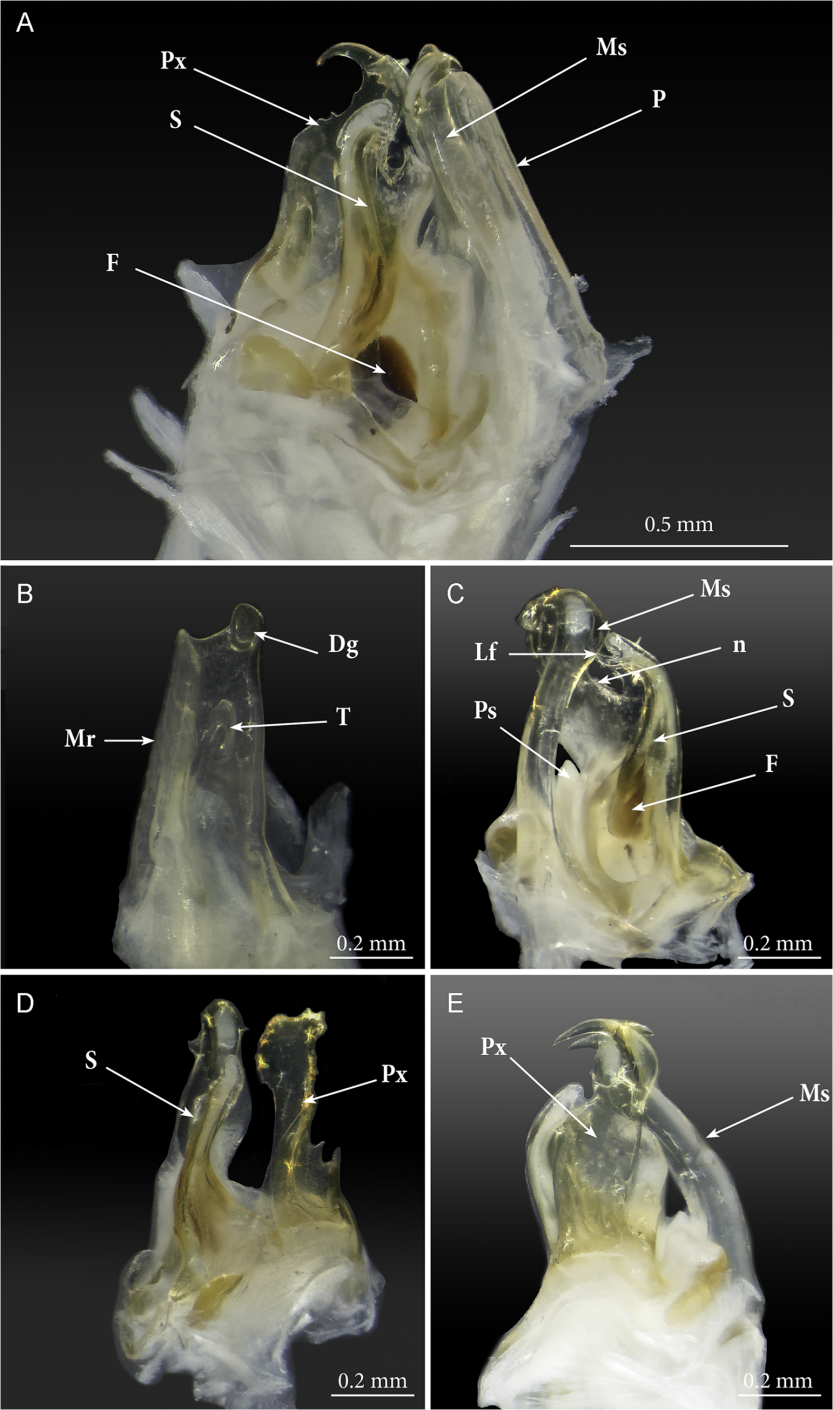
*Ommatoiulus avatar* n. sp., male paratype. Photographs of dissected gonopods showing details. (A) right gonopod, mesal view; (B) left promerite, posterior view; (C) left posterior gonopod, mesal view; (D) left posterior gonopod, posterior view; (E) left posterior gonopod, lateral view. **Abbreviations**. **Dg**. digit-shaped process; **F**. fovea; **Lf**. lamellar fold of the solenomerite; **Mr**. mesal ridge; **Ms**. mesomerite; **n**. notch; **P**. promerite; Ps. pointed process of the solenomerite; **Px**. paracoxite; **S**. solenomerite; **T**. rudimentary telopodite.

**Fig 5:**
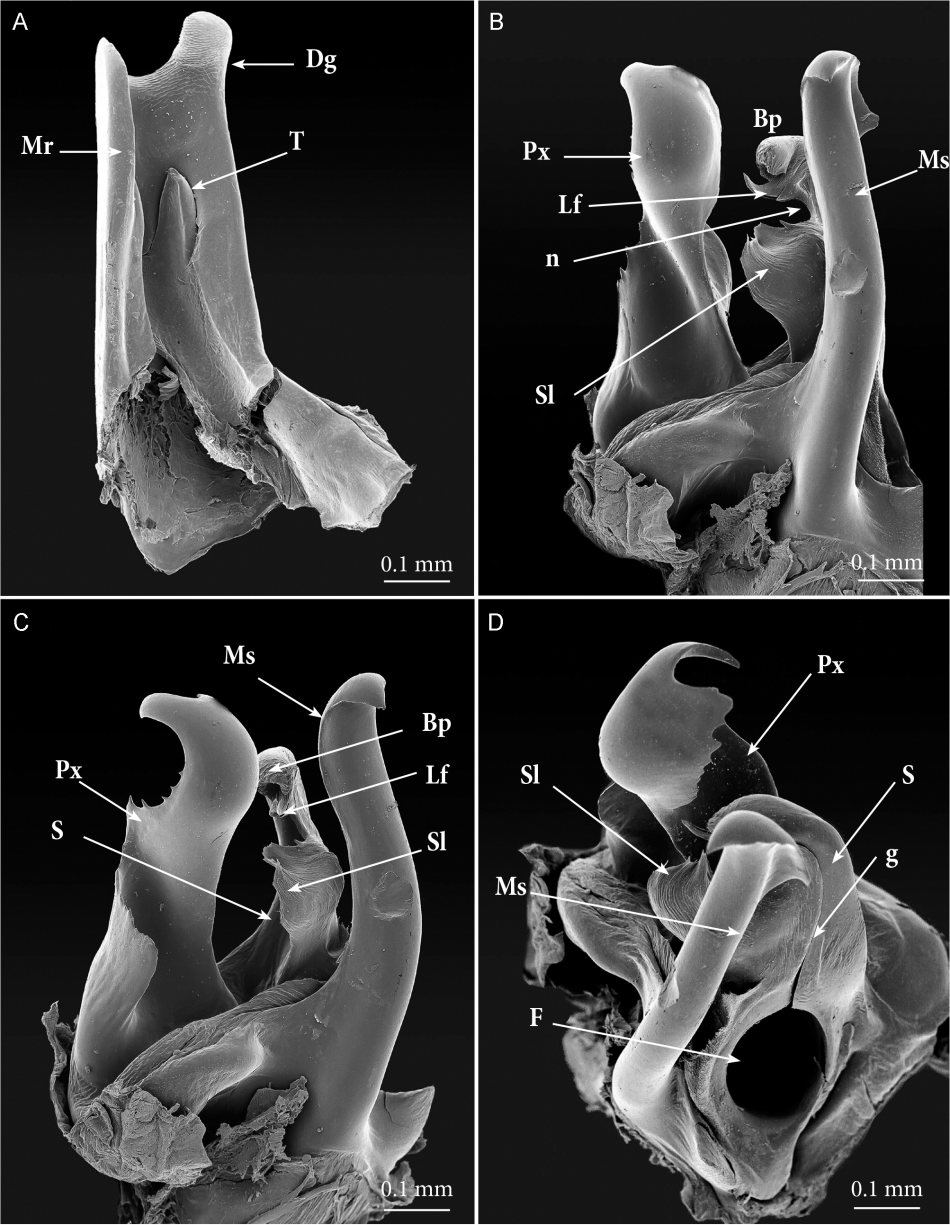
*Ommatoiulus avatar* n. sp., male paratype, gonopods details, SEM. (A) left promerite, posterior view; (B) left posterior gonopods, antero-lateral view; (C) left posterior gonopod, lateral view; (D) left posterior gonopod, meso-apical view. **Abbreviations**. **Bp**. blunt process of the solenomerite; **Dg**. digit-shaped process; **F**. fovea; **g**. seminal groove; **Lf**. lamellar fold of the solenomerite; **Mr**. mesal ridge; **Ms**. mesomerite; **n**. notch; **Px**. paracoxite; **S**. solenomerite; **Sl**. serrated lamella of the solenomerite; **T**. rudimentary telopodite.

**Fig 6:**
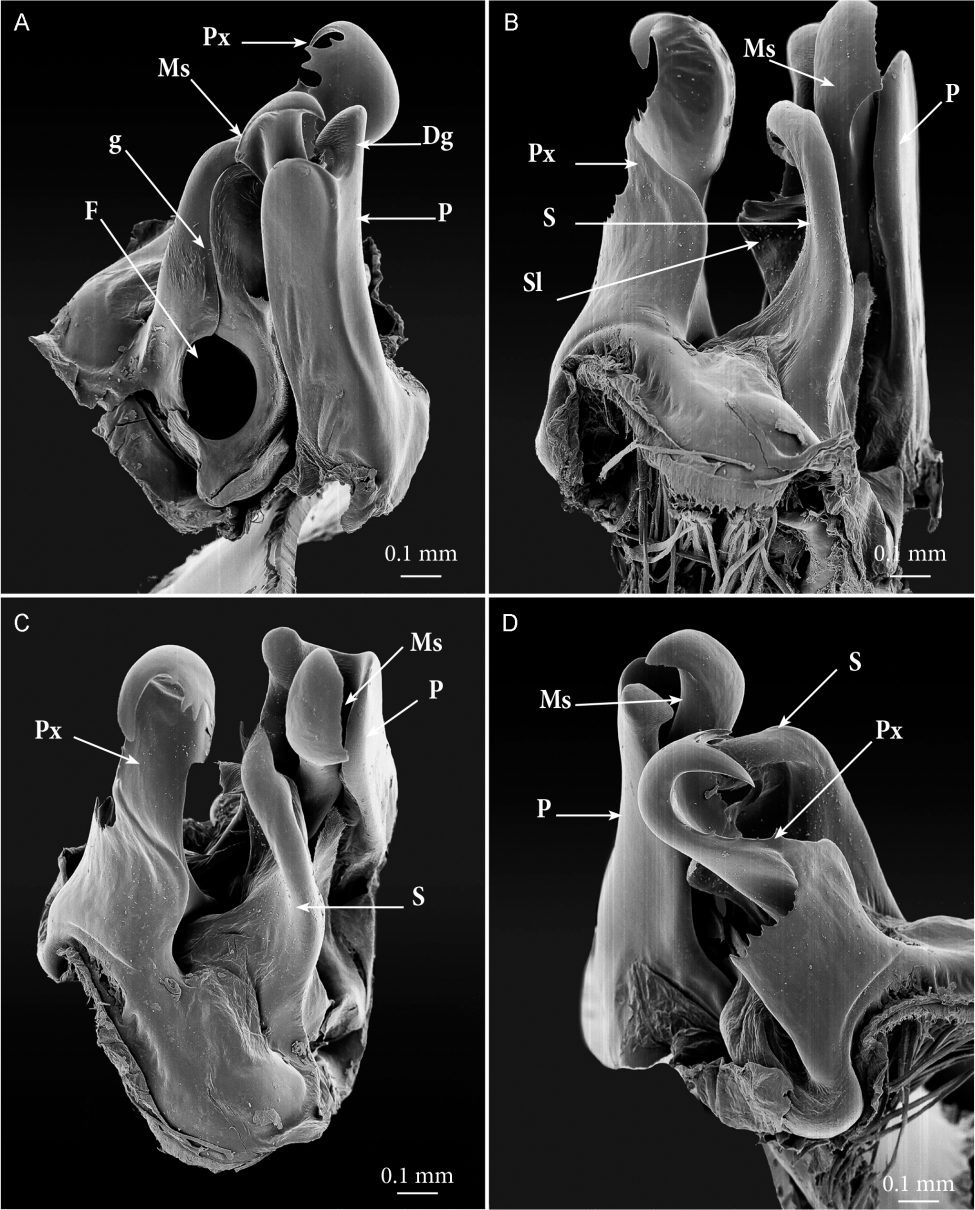
*Ommatoiulus avatar* n. sp., male paratype, gonopod details, SEM. (A) right gonopod, mesal view; (B) right gonopod, meso-posterior view; (C) right gonopod, postero-apical view; (D) right gonopod, latero-apical view. **Abbreviations**. **Dg**. digit-shaped process; **F**. fovea; **g.** seminal groove; **Lf**. lamellar fold of the solenomerite; **Ms**. mesomerite; **P**. promerite; **Px**. paracoxite; **S**. solenomerite; **Sl**. serrated lamella of the solenomerite.

**Fig 7:**
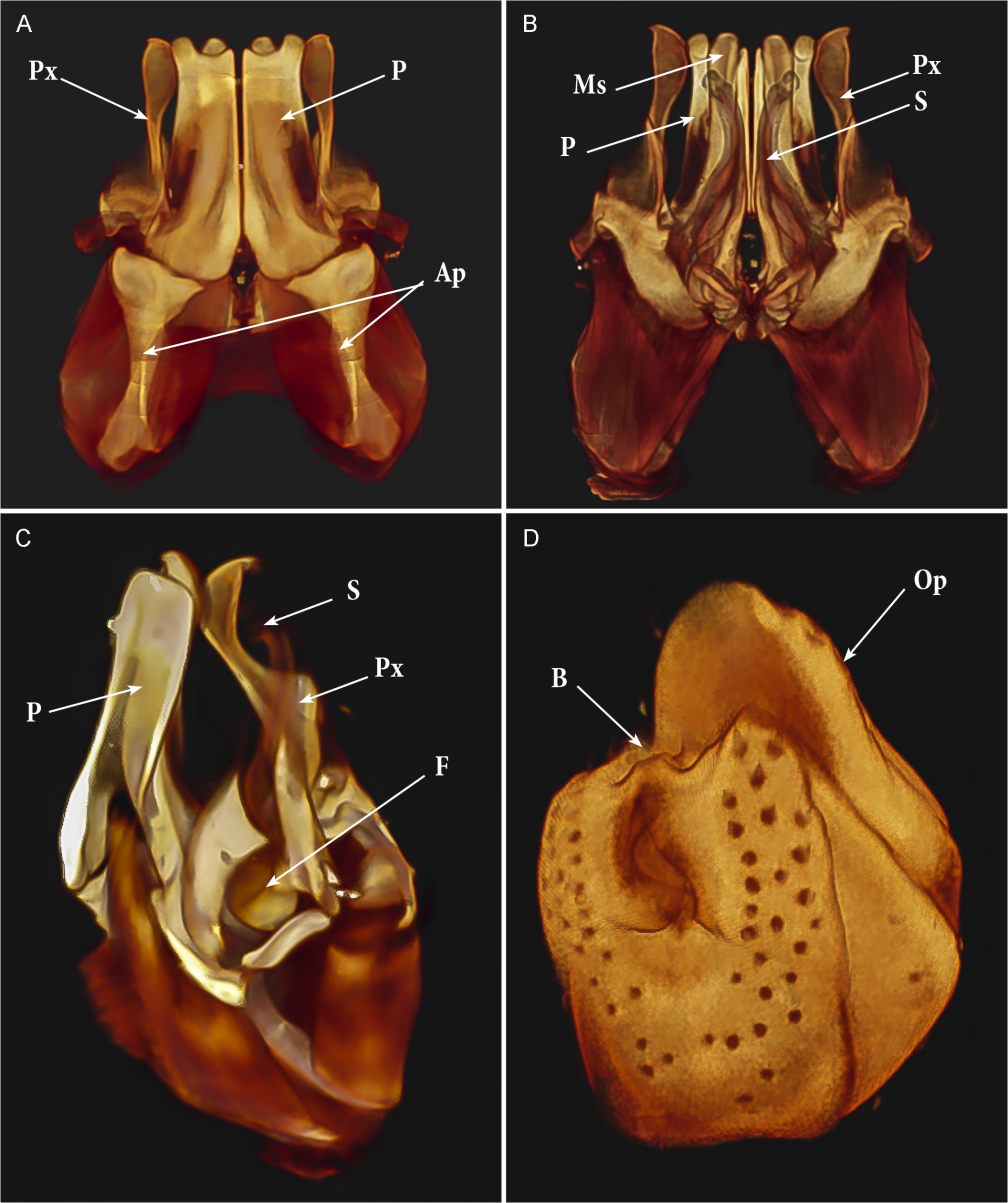
*Ommatoiulus avatar* n. sp. Volume rendering: Amira snapshots enhanced to illustrate the gonopods and left vulva in the cybertypes. (A) Gonopods, anterior view; (B) Gonopods, posterior view; (C) Left gonopod, mesal view; (D) Left vulva, posterior view. Abbreviations. **Ap**. apodemes; **B**. bursa; **F**. fovea; **Ms**. mesomerite; **Op**. operculum; **P.** promerite; **Px**. paracoxite; **S**. solenomerite. Images not to scale.

**Fig 8:**
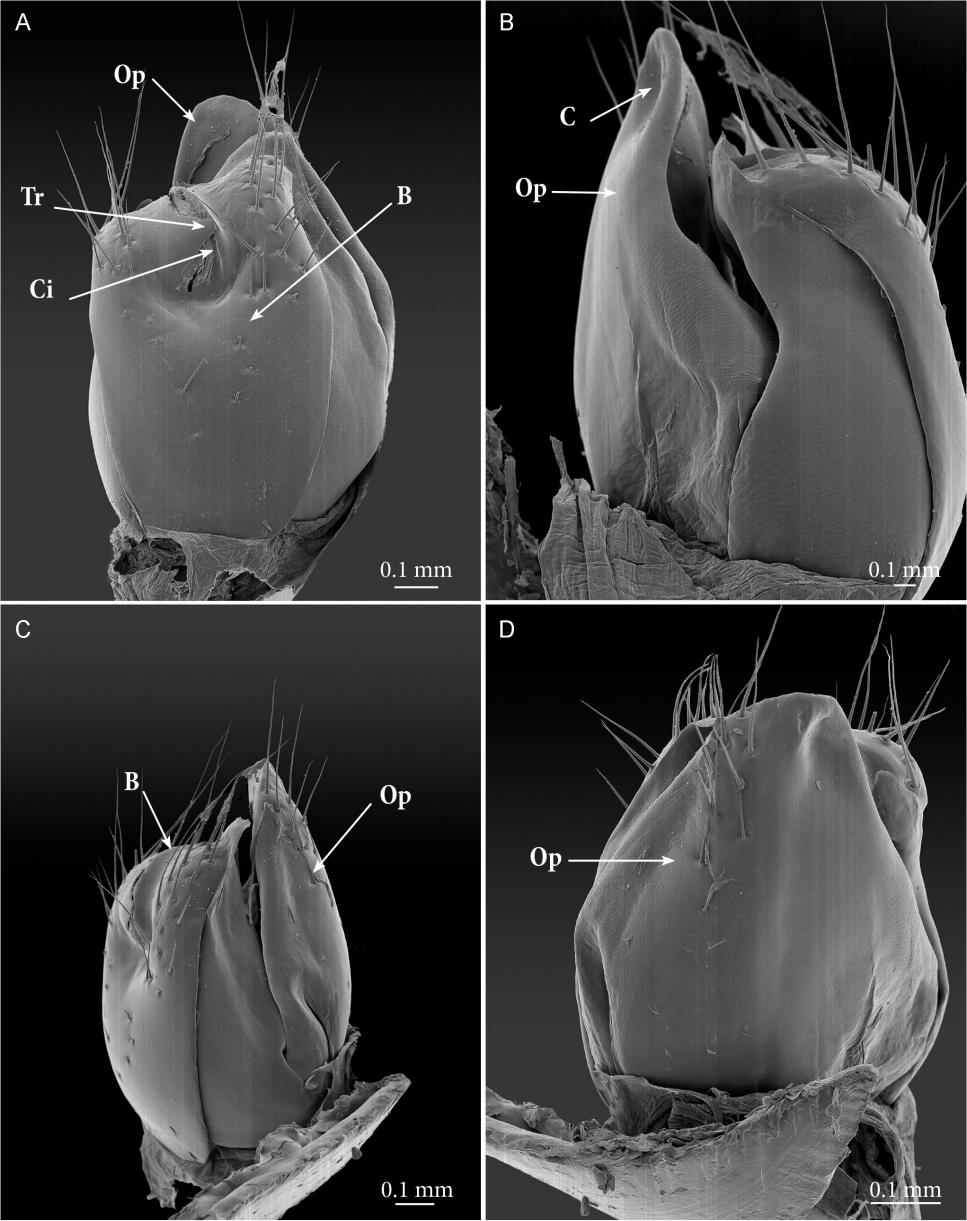
*Ommatoiulus avatar* n. sp., female paratype, vulvae details, SEM. (A) left vulva, posterior view; (B) left vulva, mesal view; (C) left vulva, lateral view; (D) left vulva, anterior view. **Abbreviations**. **B**. bursa; **C**. concavity. **Ci**. crest of the valve; **Op**. operculum; **Tr**. triangular process of the valve.

**Fig 9:**
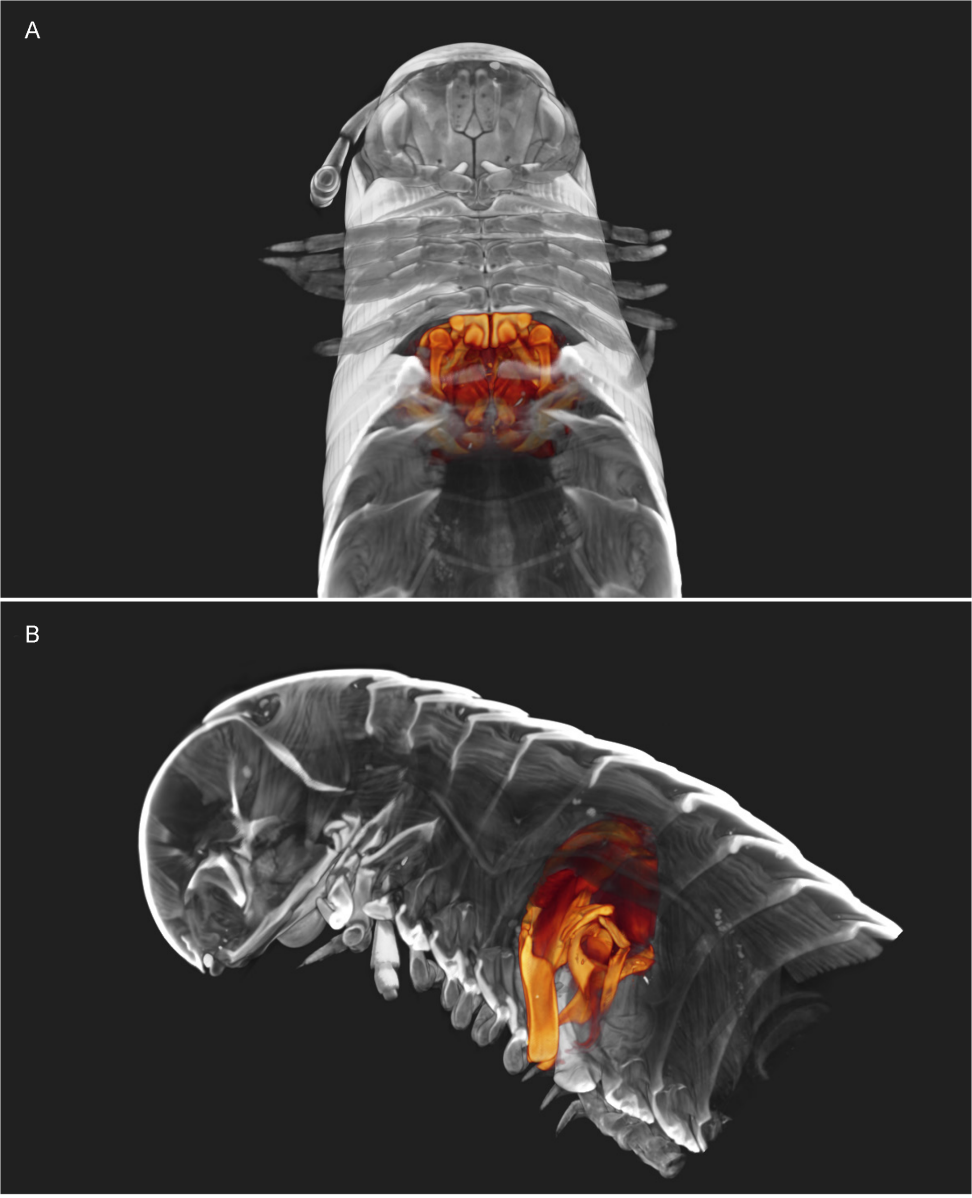
*Ommatoiulus avatar* n. sp. 3D virtual dissection of the holotype depicting the gonopods *in situ*. (A) Oblique transverse cut at body ring 10, showing attached musculature; (B) Parasagittal cut showing musculature and other anatomical structures. Images not to scale.

**Fig 10:**
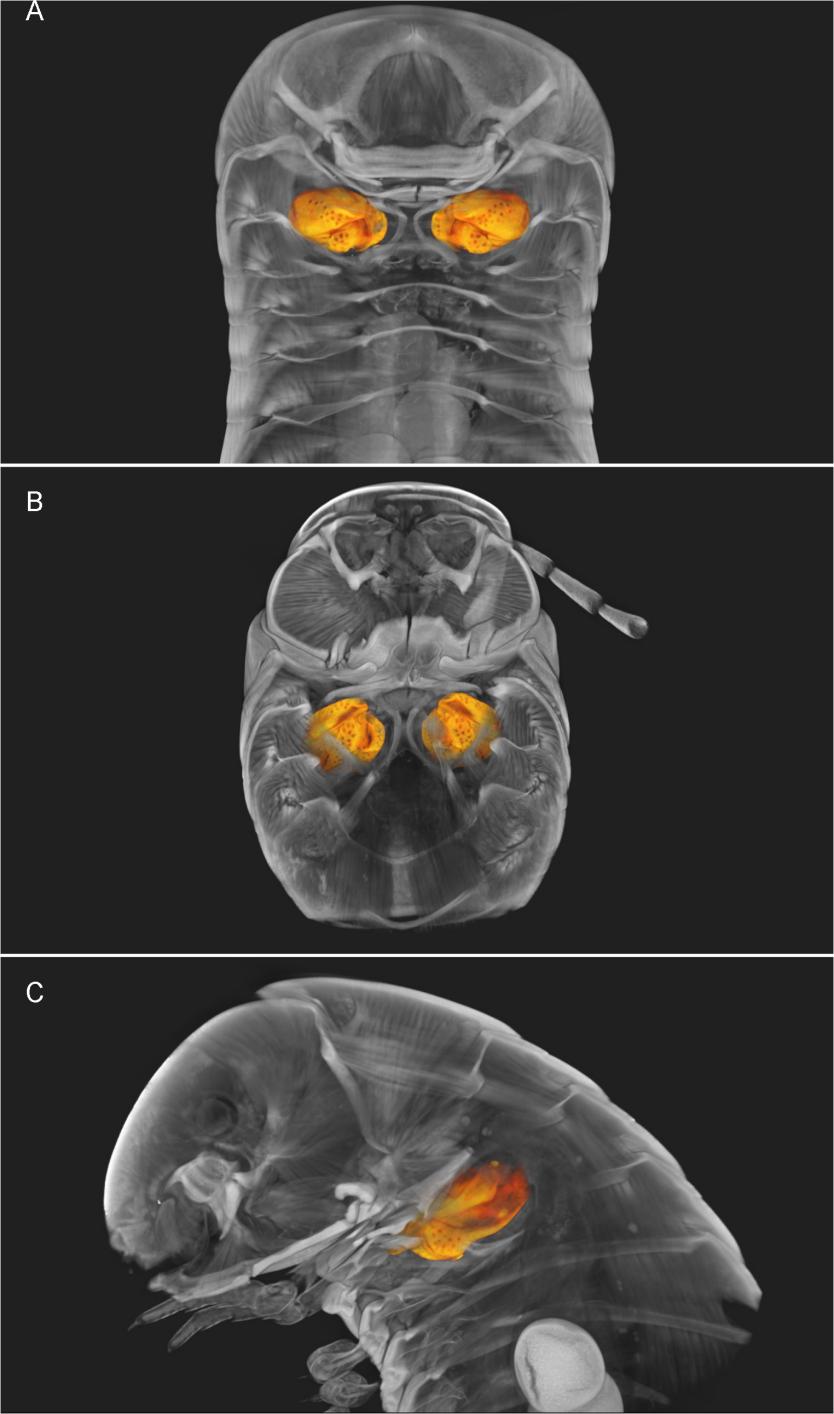
*Ommatoiulus avatar* n. sp. 3D virtual dissection of the paratype, depicting vulvae *in situ*. (A) Horizontal cut; (B) Transverse cut at body ring 3; (C) Parasagittal cut. Images not to scale.

**Fig 11:**
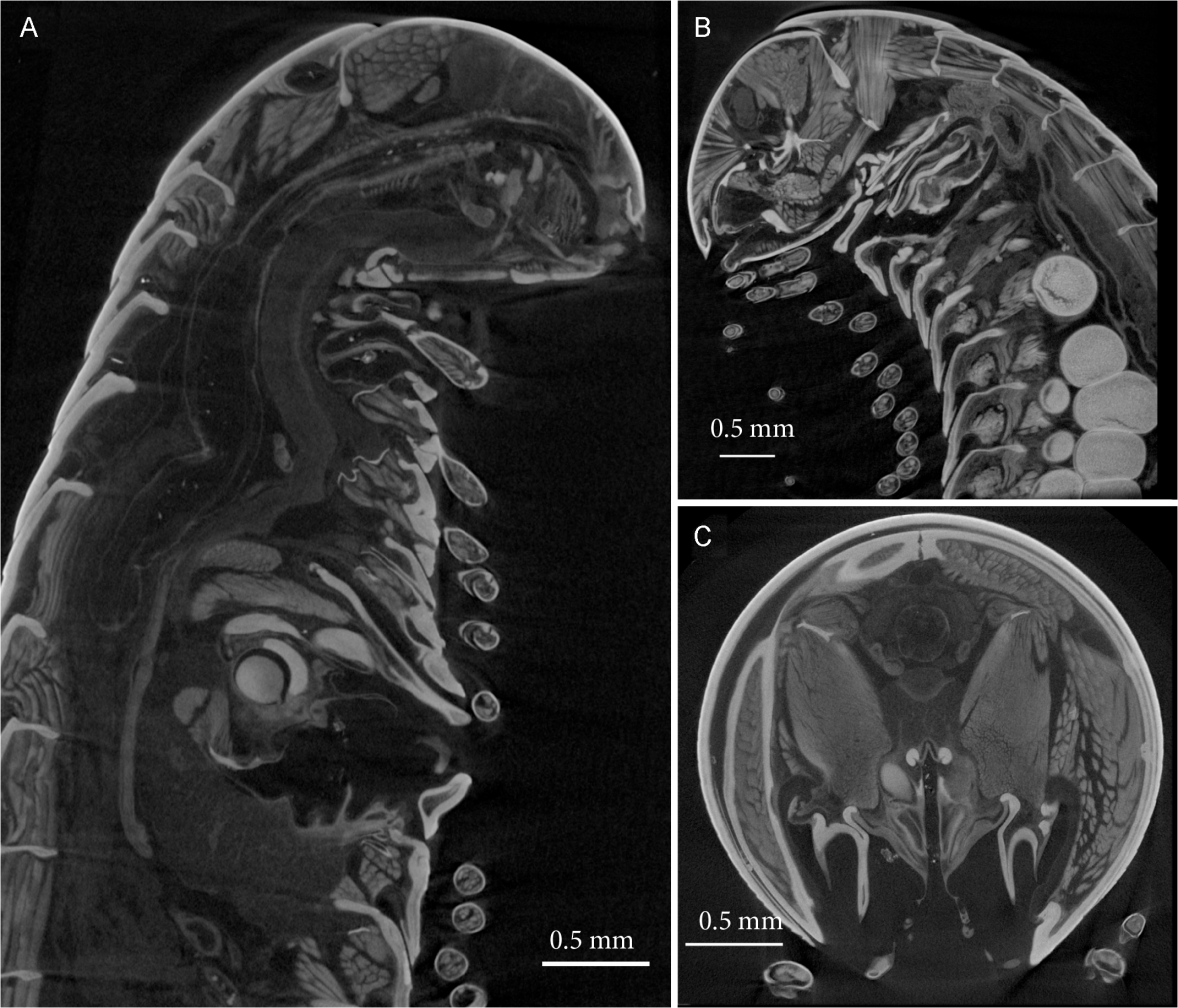
*Ommatoiulus avatar* n. sp. Virtual sections of the cybertypes (male and female), selected from the archived image stacks and displayed with unaltered window and level (brightness and contrast) settings. (A) Cybertype male, parasagittal section showing gonopods, musculature and other anatomical structures. (B) Cybertype female, parasagittal cut showing vulvae, eggs, musculature and other anatomical structures. (C) Cybertype male, transverse section showing parts of the gonopods.

**Material: Holotype**. male, SPAIN, Andalusia, Malagá, Cómpeta, Pine forest, 6.ix.2005, R.D. Kime leg. Coll. ZMUC–ZMUC00040346. **Paratypes**: 1 male, 5 females, 1 intercalary male, SPAIN, Andalusia, Malagá, Casa Lamina, Cómpeta, 15. i. 2006. R.D. Kime leg. Coll. ZMUC–ZMUC00040347; 1 male, 1 female, SPAIN, Andalusia, Malagà, Cerro Guzman, Canillas de Albaida, 10. xi. 2005, R.D. Kime leg. Coll. ZMUC–ZMUC00040348; 1 male, 1 female, 4 immatures, SPAIN, Andalusia, Malagà, Cerro Guzman, Canillas de Albaida, 22. iv. 2009, R.D. Kime leg. COll. ZMUC–ZMUC00040349; 1 female, SPAIN, Andalusia, Malagá, Cómpeta, Pine forest, 6. xi. 2005 R.D. Kime leg. Coll. NHMW–NHMW8405; 2 males, 2 sub-adult females, SPAIN, Andalusia, Malagá, Cómpeta, Pine forest, 6. xi. 2005 R.D. Kime leg. NHMW–NHMW8406; 2 males, 5 females, 2 sub-adult males, SPAIN, Andalusia, Malagá, 36°50’21. 8 N, 04°00’31.2, W, Suzanne Hill leg. Coll. NHMW–NHMW8407.

#### Material: Cybertypes

Volume image data (head + 8–10 anterior rings) of the physical types: Holotype, Coll. ZMUC–ZMUC00040346 and 1 female paratype, Coll. NHMW–NHMW8405. Contrast-enhanced microCT images with 1.9–3.4μm voxel size, archived as 8-bit grayscale PNG image stacks.

All 3D images and metadata of the physical types are deposited in Dryad (datadryad.org; doi:10.5061/dryad.2pf38), where they can be freely accessed as an electronic, universal representation of the physical type material for any subsequent taxonomic or other kind of morphological studies.

#### Diagnosis

Most similar to *Ommatoiulus bavayi* (Brölemann, 1897), with which it agrees in the shape of promerite and mesomerite and the way they fit closely together; also in the sinuous paracoxite. The two species differ, however, in the shape of the solenomerite, much broader and with a distal lamella in the new species, *vs*. with obviously narrower processes in *Ommatoiulus bavayi*. The paracoxite is much more serrated, broader and complex in the new species than in *Ommatoiulus bavayi*.

#### Etymology

In computing, an avatar is the digital representation of the user or the user's alter ego or character. The name here refers to the cybertype, which is a graphical representation of the physical holotype.

#### Description

Males: L: 25.6–33.2 mm; H: 2.1–2.9 mm, 42–46 PR+1-2AR+T. Females: L: 26–38.2 mm; H: 2.5–3.5 mm, 46–52 PR+2–3 AR+T.

General color after 6–10 years in alcohol (Fig 1) brownish with yellowish and black marbling most pronounced dorsally; prozonites light-brown to yellowish with fine black sputter; metazonites with dense black median sputter, denser at ozopores level and fading on the lateral sides; dorsum with a thin black mid-dorsal line; legs light brown. Head chestnut-brown on frontal part and towards the collum, becoming lighter toward the labral zone; labral margin and mouthparts bright reddish-brown; antennae dark brown. Prozonites with scattered oblique striae; metazonites with regular striation, suture complete, rectilinear on foremost rings, curving at ozopore level from midbody rings onward, ozopores as small rounded spots, opening in metazonites at a distance behind the suture about equal to their diameter (Fig 1).

Telson: Preanal ring yellowish-light brown with a horizontal caudal projection, bearing 2+2 setae and with a hyaline tip; subanal scale triangular and setose; anal valves setose with a marginal row of numerous short setae and a submarginal row of longer ones.

#### Male sexual characters

Mandibular stipites expanded into rounded posterior-ventral lobes, first pair of legs hook-shaped, other legs with postfemoral and tibial pads (Figs 1, 2A).

Gonopods (Figs 3–7C). Promerite (**P**) sub-rectangular, *ca*. twice as long as broad, distally with a lateral digit-shaped process (**Dg**) showing a coarse scaly surface posteriorly (Figs 4B and 5A); mesal ridge (**Mr**) broader at basis, distally protruding in a rounded lobe; rudimentary telopodite (**T**) conspicuous and placed rather distally. Posterior gonopod (Figs 4C-4E, 5B–5D and 6): Mesomerite (**Ms**) simple, longer than promerite, uniformly slender and curved at midlength, closely fitting against promerite, subapically slightly expanded and bent anteriad, apex asymmetrical with a pointed lateral tip and a serrated margin; solenomerite (**S**) broad, narrowing at mid-length, anteriorly with a triangular pointed process (**Ps**) showing in the SEM several spines and serrations (Fig 6B); distal part of solenomerite broad, composed of a posterior rounded blunt process adjacent to an acuminate lamellar fold lodging the opening of the seminal groove separated from a broad serrated lamella (**Lf**) by a rounded notch (Figs 4C, 5B and 5C); seminal groove (**g**) running posteriorly from the fovea (**F**) to the process **Lf** (Figs 5D and 6A). Paracoxite (**Px**) complex, broad and sinuous (Figs 3B, 4D, 5B and 6C) showing in a posterior view a 180° meso-posteriad twist at mid-length (Figs 3B and 4D), distal part of paracoxite strongly serrated (Figs 4D and 6D), distalmost third showing in antero-lateral view as a truncated process curved posteriad (Fig 5C); in meso-apical view clearly showing an asymmetrical apical margin with a saw-like mesal surface and a hook-like lateral process pointing posteriad (Figs 4A, 4D, 4E and 5E).

#### Female sexual characters

**Vulvae** (Figs 2B, 7D and 8). Operculum (**Op**) broad, longer than the bursa, distally narrowing into a rounded apical process bearing ca. 10 setae, anterior surface subdistally with shallow concavity (**C**). Bursa (**B**) with asymmetrical valves, each distally with ca. 16–17 setae each, and 14 additional setae on surface; lateral valve with triangular process (**Tr**) covering the ‘crest’ (**Ci,** *cimier* of French authors). *Receptaculum seminis* originating from a longitudinal depression between valves and consisting of a long apodeme branch bifurcating distally, one of the branches ending in a spherical ampulla (S8 Video).

### Distribution

Spain, Andalusia.

### Comments

The species of the large genus *Ommatoiulus* Latzel, 1884, from the Spanish Province of Andalusia were recently revised by Akkari & Enghoff, who recorded 19 species, ten of which were new to science [24]. The gonopod structures of the new species we describe here places it in a group together with several other Spanish species, viz. *O*. *albolineatus* (Lucas, 1845), *O*. *baenai* Akkari & Enghoff, 2012, *O*. *bavayi* (Brölemann, 1897), *O*. *ibericus* Cauca, 1974, *O*. *inconspicuus* (L. Koch, 1881), *O*. *kimei* Akkari & Enghoff, 2012, *O*. *hoffmani* Akkari & Enghoff, 2012, *O*. *jaenensis* Akkari & Enghoff, 2012, *O*. *niger* (Attems, 1952), *O*. *recueroi* Akkari & Enghoff, 2012, and *O*. *reipi* Akkari & Enghoff, 2012. All these species share a number of morphological characters:

- Promerite with a broad folded mesal ridge and a lateral digit-shaped process
- Mesomerite slender, curved at mid-length, slightly expanded distally and bent anteriad
- Mesomerite + promertite fitting tightly together, forming a ‘pro-mesomerital forceps’
- Solenomerite broad with a distal lamella
- Paracoxite large, displaying varied degrees of complexity.

### Description of the cybertypes

This is the first new species description to be based partly upon and presented with a virtual representation (an avatar) of the holotype and paratype specimens. The cybertypes comprise two high-resolution contrast-enhanced 3D tomographic images of the holotype (male) and one 3D image of a female paratype. Because all the important taxonomic characters are found in the anterior region (head through the 7^th^ body ring), the whole anterior portions of the animals were scanned and reconstructed with 2.5μm voxels, while the gonopod region of the holotype was scanned and reconstructed with 1.7μm voxels. The volume images are presented as stacks of 8-bit PNG images, which can be read by a variety of free and commercial programs. The images were down-sampled slightly to make each folder just under 1 GB in size, giving slightly larger voxels but with no noticeable loss in image quality. The archived images have the pixel sizes and slice spacings noted below (isotropic voxels). They were archived as ZIP files for easier downloading, but owing to the efficiency of the PNG image format, the ZIP files are in fact no smaller than the original folders. The three image stacks archived in Dryad (doi:10.5061/dryad.2pf38) are as follows:

**O-avatar_holotype_I2E_3-0um_PNG.zip** (993 MB): Male (holotype), anterior part; parasagittal sections (pixel size and slice thickness = 3.0 μm)

**O-avatar_holotype_gonopods_I2E_1-9um_PNG.zip** (979 MB): Male (holotype), genital region, frontal (transverse) sections (pixel size and slice thickness = 1.9 μm)

**Ommatuoilus_avatar_female_I2E_3-4um_PNG.zip** (1.0 GB): Female (paratype), anterior part; parasagittal sections (pixel size and slice thickness = 3.4 μm)

We illustrate the cybertypes with still images and movies of volume renderings made in Amira 5.6 (Figs 2, 7, 9 and 10) and with virtual sections from the stacks (Fig 11). It is noteworthy that the images are rendered and displayed with important structures (gonopods, vulvae) highlighted as different-colored sub-volumes and not as surface renderings as has been customary in the literature. Surface renderings require a set threshold value to establish the location of the surface along each (3D) brightness (or density) gradient, yielding a two-dimensional surface within the 3D volume image, and this is usually displayed as an opaque glossy or matte object with lighting and shading chosen to emphasize the features of interest. A more complete and more meaningful visualization results from displaying not the surface of the selected (segmented) sub-volume, but the actual volume image data with opacity adjusted to reveal important details. Thus volume-rendered illustrations can be an invaluable complement to surface-rendered tomography and SEM images.

S1 Video shows a volume rendering of the anterior part of the male holotype, from a microCT scan made before staining. S2 Video is a volume rendering made from a microCT scan of the same specimen after iodine staining (the cybertype image data), with transparency adjusted to allow visualization of the secondary sexual characters (excluding the penis) i.e., genitalia (gonopods), first pair of legs and expanded lobes of stipites *in situ* while rotating the specimen (see also Figs 2A, 9 and 11A). S3–S5 Videos represent 3D models of the gonopods of the same cybertype, segmented and virtually isolated by discarding the rest of the volumetric information. These videos clearly show the various structures of the anterior and posterior gonopods, the musculature and the anterior and posterior apodemes, respectively (see also Fig 7A–7C). S5 Video allows the visualization of further details of the posterior gonopods through virtual lateral sectioning until the median plane, depicting the mesal view. The gonopods were not dissected and are displayed in their natural position and anatomical setting, revealing further information on the related musculature and the internal organs (see also Figs 9 and 11). S4 Video clearly displays the anterior and posterior apodemes of the gonopods (see also Fig 7A), structures depicted in earlier descriptions of congeneric species, but whose taxonomic value has been very poorly surveyed since then. Structures such as right and left gonopod connectors are also clearly displayed (see Fig 7B).

S6–S8 Videos illustrate the female cybertype, highlighting the vulvae and eggs *in situ* (see also Figs 2B, 10 and 11B), while fading the rest of the structures and displaying a rotating view of the sample. Additionally, a virtual sectioning of the vulvae has been displayed (S8 Video) to depict their inner structures including the spherical *receptaculum seminis*, which is seen as a white spot near the bottom of the vulva.

## Discussion

### Virtual specimens and new dimensions in myriapod taxonomy

Although myriapods are still regarded as a poorly understood taxonomic group [43], the taxonomy of myriapods has enjoyed tremendous progress and has benefited from various innovative approaches during the past few years. Among the main breakthroughs is the use of next-generation molecular methods and advanced imaging techniques. While phylogenomics has provided new insights on millipede phylogeny and has helped to resolve conflictual taxonomic situations (e.g. [2], [3]), innovative imaging techniques have opened new horizons for cybertaxonomic morphology applications [4], [5]. Just as transcriptomic and full genomic data are helping to delineate species and higher taxa while answering questions related to their phylogenetic relationships, high-detail whole-sample imaging constitutes a new and powerful tool to generate and share voluminous and three-dimensional morphological/anatomical datasets in high resolution, further stimulating the taxonomic, phylogenetic and any other morphological studies on these taxa.

Among recent examples, synchrotron and lab-based X-ray microtomography have been used to describe extinct myriapods from amber fossils or to depict new phylogenetic characters in extant species (e.g. [32], [44], [45]). The first 3D representation published with a new myriapod species description was for the centipede *Eupolybothrus cavernicolus* [5], but the 3D images were not used to inform the species description or to illustrate any particular details or structures. Blanke and Wesener provided the first 3D reconstruction of the head of the millipede *Brachycybe lecontii*, highlighting some details of its internal anatomy [32]. In contrast, the present description of the new millipede species *O*. *avatar* is the first for which microCT images have been used for describing and depicting the characters important for the taxonomy of the group and the first to be accompanied by published cybertypes of the type material.

The chief advantage of microCT is the ability to produce complete, size-calibrated, fully aligned volume images of intact specimens, which are not damaged by the processing. Contrast staining with iodine imparts clear X-ray contrast to the non-chitinous tissues in arthropods [41], [46] and is easily removed with 70–90% ethanol, a common storage medium for arthropod specimens. With this treatment, it was not necessary to critical-point dry the samples or to use a chemical desiccant like HMDS, and the type material can be returned to the museum undamaged. The type material studied here remained intact and no dissection was performed to depict the various genital and adjust structures used for species description. The gonopods and vulvae were displayed in their natural positions and anatomical settings. The musculature and the connection of the genitalia to the internal organs are also clearly displayed. Further virtual sections allowed visualising further inner structures of the vulvae and details of the gonopods. The unaltered material was returned to the museum’s collection in 70% ethanol, demonstrating the potential of microCT in studying unique and even fragile museum specimens.

The resolution of a tomographic image is limited by the reconstructed voxel size, which is in turn a fixed fraction of the field of view: thus there is a direct trade-off between imaged sample size and achieved resolution. The maximal resolution of a microCT image is not always sufficient to reveal minute surface structures which are better visualised with SEM. The use of multiple types of images, e.g. light photography, digital drawing, SEM, and others to enhance taxonomic descriptions has been acknowledged on several occasions, e.g. [4], [29], [47], among others.

Microtomographic images are particularly useful in describing new millipede species, because the most important discriminating characters are in the male gonopods, which in most cases cannot be examined without dissection. Contrast-enhanced microCT imaging also opens new possibilities for exploiting soft-tissue characters for taxonomic descriptions, phylogenetic studies (e.g. [48]), as well as for functional morphology studies [46].

### Cybertypes

The idea of a cybertype–a digital simulacrum of a physical type specimen–has been discussed extensively in the recent literature and at a number of international meetings (see especially [37] and references therein). As a supplement to the biological material, a cybertype adds value to the material collections and facilitates sharing of primary biodiversity data, reducing the reliance on handling of physical specimens to allow a new species to be included in more research efforts. While we emphasize here the morphological cybertype, we recognize that other kinds of adjunct data can and should be included with the characterization of a new species whenever appropriate.

In a multifaceted description of a new centipede species, Stoev et al. included a full transcriptome, a DNA barcode, a movie of the living animal, and a microCT image of a paratype [5]. Their report referred to the total set of digital data for the new species as its cybertype, and this set an example for how much more complete a new species description can be by including more than the morphological description of defining characters. However, the microCT images did not contribute to the species description, and the quality of the archived image data is likely not sufficient for further research on the species.

With the description of *O*. *avatar*, we have presented a cybertype comprising high-resolution images of the intact and vouchered type material. The cybertype images were employed in the species description and are supplemented with special volume-rendered images of the genitalia, critical structures for recognizing millipede species. Our morphological cybertype also meets the criteria set by [37: 4], namely anatomical fidelity, connection with the physical type material, and open accessibility. The 3D images are deposited at Dryad (doi:10.5061/dryad.2pf38).

### Myriapods and cybertaxonomy

With the recent significant advances in disseminating taxonomic knowledge, myriapods have become a cyber-mediated group. Information on myriapods is now accessible through several web portals, online databases and libraries as well as in varied archives allowing data storage. Gene sequences and COI barcodes of many taxa are available in GenBank (http://www.ncbi.nlm.nih.gov/genbank), and images illustrating species and related features have become available in Morphbank (http://www.morphbank.net/).

Online databases like Chilobase (http://chilobase.bio.unipd.it/) and Fauna Europaea (http://www.faunaeur.org/) provide catalogues of all known Chilopoda and European Diplopoda respectively, offering consistent taxonomic information, updated nomenclature for all species, and information on their distribution [49], [50]. In this same context, geo-referenced information, e.g. GBIF (http://www.gbif.org/), as well as its derivatives Edaphobase (http://portal.edaphobase.org/) and GloMyrIS (http://www.gbif.de/node/151), now offer free access and unlimited downloads for all records published via their network, which can be explored and sorted by occurrences, species, datasets, and countries.

Interactive identification keys for myriapod taxonomy are a new and engaging tool born of the cybertaxonomic era. Since the work of Linnaeus in 1758 [51], identification keys have been a vital tool in taxonomy and continue to be part of most recent contemporary revisions and taxonomic treatments. They are also widely used by amateur naturalists. However, with today’s rapidly developing media, a well-designed and ‘dynamic’ key with a clear and user-friendly interface may have greater potential for facilitating species identification and for adoption by a much broader user base. Most emerging interactive keys offer a tremendously improved graphic material, an unrestricted character use and in most cases leaving a minimal margin to error (see [52]). Among the rare examples for Myriapoda, we cite the interactive key for the centipede genus *Eupolybothrus* made with DELTA software [53], and more recently, the highly visual and intuitive key presented for a group of species of the millipede genus *Ommatoiulus*, developed with the Flash software (http://zookeys.pensoft.net/export.php_files/5763-G-1-layout_key.html). The key included over 300 different images varying from line drawings to photographs, microphotographs, SEM and rotational-SEM to illustrate the different species and the varied structures used to distinguish them [4]. Another noteworthy example is ChiloKey (http://www.interactive-keys.eu/chilokey/default.aspx), a matrix-based interactive key, which aims at the identification of 179 species of European Geophilomorpha [7].

In myriapodology, the cybertaxonomic applications are not limited to interactive keys but encompass such initiatives as the cybertaxonomic revision of genus *Eupolybothrus* by Stoev et al. which promoted innovative publishing methods through tagging enhancement, text and data processing, and inserting links to BOLD, MorphBank, Google Earth maps, and an interactive key for the genus [53]. On the same note, Stoev et al. in 2013 introduced an example of a holistic species description in a recent study on the same genus, combining conventional taxonomic treatment, transcriptome sequencing data, DNA barcode and microCT data [5]. All the data were stored in the repositories GigaDB (the complete database), NCBI (COI and transcriptome sequences), BOLD (COI), Morphbank (morphological images), Morphosource (microCT raw data, 3D model), Plazi (taxon treatments of legacy literature related to genus *Eupolybothrus*). In the revision of genus *Ommatoiulus* of Tunisia, Akkari et al. introduced a novel way of visualizing millipede genitalia, based on SEM images imported into Adobe Flash CS5 and combined to form a rotating animation readable within the PDF [4].

The species description presented here is another example of how the practice of taxonomic science need no longer be seen as quaint and old fashioned, but as a discipline that reflects the ways that knowledge is produced, shared, and used in our modern era. New endeavors and current technologies will certainly continue to play important roles in that, and the spirit of sharing data will make it only go forwards. Cybertypes will serve well in this context, but are never meant to actually replace the physical type specimens.

## Supporting Information

S1 Video S1-Omma_avatar_unstained_5-0um.mp4. *Ommatoiulus avatar* **n. sp.** male cybertype, unstained. Volume rendering of microCT image.
(MP4)

S2 Video S2-Omma_avatar_male_whole-rotate. mp4. Ommatoiulus avatar n. sp. male cybertype, iodine stained, with secondary sexual characters in situ highlighted in volume rendering.
(MP4)

S3 Video S3-Omma_avatar_male_gonopods1.mp4. *Ommatoiulus avatar* **n. sp.** male cybertype, gonopods segmented in Amira and displayed as a rotating volume rendering, showing apodemes.
(MP4)

S4 Video S4-Omma_avatar_male_gonopods2.mp4. *Ommatoiulus avatar* **n. sp.** male cybertype, gonopods in rotating volume rendering.
(MP4)

S5 Video S5-Omma_avatar_male_gonopods-deslice.mp4. *Ommatoiulus avatar* **n. sp.** male cybertype, gonopods, virtual sectioning of a volume rendering.
(MP4)

S6 Video S6-Omma_avatar_whole-female_slice-deslice.mp4. *Ommatoiulus avatar* **n. sp.** female cybertype, vulvae in situ, lateral sectioning of volume rendering.
(MP4)

S7 Video S7-Omma_avatar_female_fade-rotate.mp4. *Ommatoiulus avatar* **n. sp.** female cybertype, vulvae and oocytes *in situ*, volume rendering with fading effect and rotating view.
(MP4)

S8 Video S8-Omma_avatar_female_vulva-slice.mp4. *Ommatoiulus avatar* **n. sp.** female cybertype, one vulva, sectioning of volume rendering.
(MP4)
